# Orthodontic Biomechanical Reasoning with Multimodal Language Models: Performance and Clinical Utility

**DOI:** 10.3390/bioengineering12111165

**Published:** 2025-10-27

**Authors:** Arda Arısan, Celal Genç, Gökhan Serhat Duran

**Affiliations:** 1 Independent Researcher, Ankara 06510, Turkey; 2 Department of Orthodontics, Faculty of Dentistry, Çanakkale Onsekiz Mart University, Çanakkale 17100, Turkey; celalgenc@comu.edu.tr (C.G.); gokhanserhat.duran@comu.edu.tr (G.S.D.)

**Keywords:** orthodontics, orthodontic biomechanics, multimodal large language models, biomechanical reasoning, clinical decision support, AI in dental engineering

## Abstract

**Background**: Multimodal large language models (LLMs) are increasingly being explored as clinical support tools, yet their capacity for orthodontic biomechanical reasoning has not been systematically evaluated. This retrospective study assessed their ability to analyze treatment mechanics and explored their potential role in supporting orthodontic decision-making. **Methods**: Five publicly available models (GPT-o3, Claude 3.7 Sonnet, Gemini 2.5 Pro, GPT-4.0, and Grok) analyzed 56 standardized intraoral photographs illustrating a diverse range of active orthodontic force systems commonly encountered in clinical practice. Three experienced orthodontists independently scored the outputs across four domains—observation, interpretation, biomechanics, and confidence—using a 5-point scale. Inter-rater agreement and consistency were assessed, and statistical comparisons were made between models. **Results**: GPT-o3 achieved the highest composite score (3.34/5.00; 66.8%), significantly outperforming all other models. The performance ranking was followed by Claude (57.8%), Gemini (52.6%), GPT-4.0 (48.8%), and Grok (38.8%). Inter-rater reliability among the expert evaluators was excellent, with ICC values ranging from 0.786 (Confidence Evaluation) to 0.802 (Observation). Model self-reported confidence showed poor calibration against expert-rated output quality. **Conclusions**: Multimodal LLMs show emerging potential for assisting orthodontic biomechanical assessment. With expert-guided validation, these models may contribute meaningfully to clinical decision support across diverse biomechanical scenarios encountered in routine orthodontic care.

## 1. Introduction

In recent years, Machine Learning (ML) has attracted the attention of researchers in orthodontics by offering potential solutions to various clinical challenges. While early applications focused on tasks such as automated cephalometric analysis [1,2,3], more recent studies have expanded the role of ML models to include treatment planning [4,5,6], dental segmentation across various imaging modalities [7,8], and facial esthetics prediction [9,10,11]. These systems aim to assist orthodontists by automating workflows, improving diagnostic precision, and reducing inter-operator variability. These models have also been investigated for their potential to detect dental anomalies [12], predict extraction decisions [4,5], and identify conditions such as plaque and gingivitis from intraoral photographs [13,14,15].

Successful orthodontic treatment often depends on patient cooperation, particularly when appliances require active participation. To enhance engagement and adherence, AI-based approaches have also been investigated as promising tools for addressing behavioral challenges such as compliance and communication in healthcare [16]. One possible application is AI-driven remote monitoring, where patients capture intraoral images using smartphones during treatment. This approach has demonstrated potential across various modalities, including fixed appliances [13] and clear aligners [17]. Remote monitoring has been shown to improve therapy adherence in other medical fields and has been positively received by both patients and dental practitioners [14,18].

However, interpreting intraoral photographs is inherently challenging for AI systems due to variations in lighting, angulation, soft tissue interference, and tooth morphology. These baseline complexities become even more pronounced in the presence of active orthodontic mechanics such as brackets, arch wires, or elastics. Even in their absence—for example, in plaque detection or Angle classification from clean images—model performance has often remained below optimal levels, indicating that current limitations extend beyond the presence of mechanics. For instance, one study reported only 69% accuracy in distinguishing Class II from Class I cases using a single-photo mobile application [19]. Another study found that although ML models outperformed orthodontists in Angle classification from photographs, their performance in estimating overjet and overbite was suboptimal, with mean absolute errors of 1.98 ± 2.11 mm and 1.28 ± 1.60 mm, respectively [20]. Furthermore, even AI systems designed for remote oral hygiene monitoring during orthodontic treatment have shown low sensitivity and moderate accuracy, often underreporting issues such as plaque and gingivitis [14].

Recent advances in Multimodal Large Language Models (LLMs), a specialized subset of Machine Learning, have enabled AI systems to process not only textual but also complex visual inputs, including high-resolution intraoral photographs [21,22]. When accessed through publicly available application programming interfaces (APIs), these models offer significant potential in orthodontics, where visual information plays a central role in clinical decision-making. Their potential extends beyond orthodontics to the wider dental field, as highlighted by Puleio et al. [23], who systematically reviewed the clinical, research, and educational applications of ChatGPT (versions 3.5 and 4) and related LLMs in dentistry. The authors reported that generative and multimodal models are increasingly being adopted for diagnostic support, patient communication, academic writing, and teaching. In orthodontics, they emphasized the importance of structured, domain-specific prompting to improve reliability and interpretability of AI-generated outputs. Incorporating such cross-disciplinary evidence underscores the broader relevance of LLMs within dental innovation and provides contextual grounding for the present study. LLMs can assist clinicians by directly interpreting biomechanical features from image content alone, without requiring patient history, demographic data, or radiographs. This capability may help identify orthodontic appliances, evaluate active treatment mechanics, and detect errors in force application. As a result, structured model outputs have the potential to enhance diagnostic consistency, reduce inter-examiner variability, and support less experienced practitioners in making more accurate clinical decisions.

Determining whether LLMs can reliably interpret standardized intraoral images with active orthodontic appliances is an essential step toward integrating AI into clinical workflows. In this study, we implemented a straightforward and reproducible evaluation framework by submitting structured prompts directly to LLMs via public APIs, without the need for custom-built interfaces or system-level integration. Although APIs are widely used in other domains, their structured application in orthodontics remains rare. To our knowledge, no prior study has systematically used this approach to evaluate biomechanical reasoning in LLMs. Our method not only benchmarks model performance but also offers a clinically relevant and accessible way to explore real-world applications, including remote monitoring and decision-support systems [8,24,25]. While LLMs are intended to complement rather than replace clinical expertise, their implementation must be carefully validated and regulated to avoid unsupervised recommendations. Ultimately, orthodontists’ clinical judgment remains central to safe and effective care [26].

The aim of this study was to systematically evaluate the biomechanical reasoning capabilities of five publicly available multimodal large language models in interpreting standardized intraoral photographs containing active orthodontic appliances, using a structured prompt-response protocol and blinded expert scoring.

## 2. Materials and Methods

### 2.1. Dataset

Written informed consent was obtained from all patients at the time of treatment. This retrospective study was conducted in accordance with the Declaration of Helsinki and approved by the Non-Interventional Clinical Research Ethics Committee of Çanakkale Onsekiz Mart University (protocol code 2025-218).

A total of 56 standardized intraoral photographs depicting active orthodontic mechanics were included, encompassing cases across the deciduous, mixed, and permanent dentition stages. The dataset composition reflected the diversity of clinical orthodontic practice and is summarized in Table 1. Of the 56 photographs, 32 (57.1%) depicted permanent dentition, 18 (32.1%) showed mixed dentition, and 6 (10.7%) represented deciduous dentition. Regarding malocclusion classification, 24 cases (42.9%) were Class I, 21 cases (37.5%) were Class II, and 11 cases (19.6%) were Class III. Appliance types included conventional fixed appliances with stainless steel brackets (n = 38, 67.9%), ceramic brackets (n = 8, 14.3%), self-ligating brackets (n = 6, 10.7%), and removable appliances (n = 4, 7.1%).

All images were captured under controlled clinical conditions using a DSLR camera with macro lens and studio lighting and were converted from RAW to high-resolution JPEG format. Only photographs conforming to the American Board of Orthodontics (ABO) guidelines for angulation and orientation were included.

**Figure 1:**
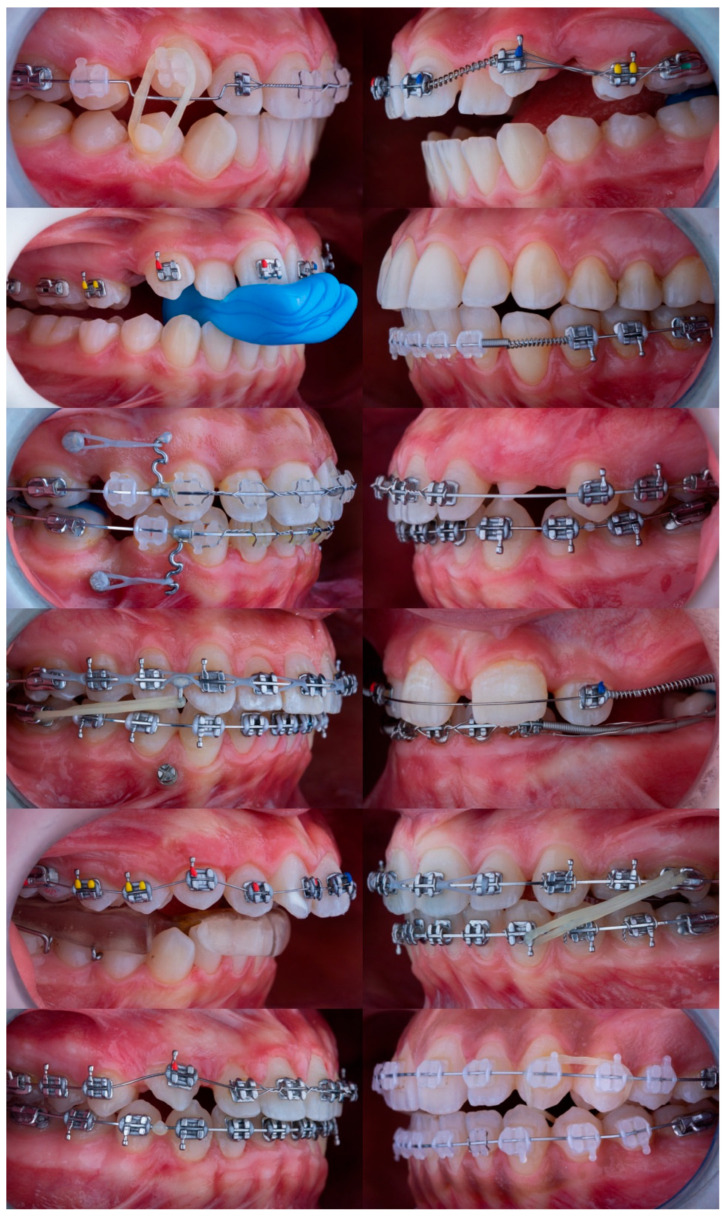
Representative sample of 12 standardized intraoral side-view photographs from the full dataset (N = 56). The images display a variety of orthodontic appliances and biomechanical configurations used to evaluate model performance.

Sample size estimation using G*Power 3.1 indicated that at least 28 images would be required to detect statistically significant differences in a repeated-measures ANOVA design. The calculation was based on the following parameters: effect size f = 0.25 (medium effect), α error probability = 0.05, power (1 − β) = 0.80, number of groups = 5 (corresponding to the five evaluated models), number of measurements = 4 (corresponding to the four evaluation domains: observation, interpretation, biomechanics, and confidence evaluation), and correlation among repeated measures = 0.5 (moderate correlation assumption). The final dataset of 56 photographs exceeded this threshold, ensuring sufficient statistical power for detecting meaningful differences among models [27,28,29]. Representative examples from the dataset are shown in Figure 1.

### 2.2. Large Language Models

Five publicly available multimodal large language models (LLMs) were evaluated between March and April 2025 through their official API-based platforms. The evaluated models were GPT-4.0 and GPT-o3 (OpenAI, San Francisco, CA, USA), Claude 3.7 Sonnet (Anthropic, San Francisco, CA, USA), Gemini 2.5 Pro (Google DeepMind, Mountain View, CA, USA), and Grok (xAI, San Francisco, CA, USA). All models were accessed in their default configuration via official API endpoints, without any additional training, fine-tuning, or custom data integration. Each model was queried independently with identical prompts and identical image inputs to ensure consistency across evaluations.

To ensure full reproducibility, detailed technical specifications for all model evaluations—including exact model versions, API endpoints, evaluation dates, and parameter settings—are summarized in Table 2. All models were accessed through standard commercial API subscriptions, without access to research-grade versions or preferential tiers.

### 2.3. Prompt Design and Output Structure

Each of the 56 standardized intraoral photographs was analyzed by the five multimodal LLMs. The models generated their assessments exclusively from the visual content of the images, without access to demographic data, clinical history, or radiographs. To ensure consistency, all models were queried with an identical JSON-based structured prompt. For each model, all 56 images were evaluated within a concentrated time window to minimize version drift. Each image was evaluated in a fresh session to avoid contextual carry-over. Each query was initialized in a fresh session to avoid contextual carry-over and potential bias between cases. This predefined output schema contained four fields. Observation referred to the identification of anatomical and orthodontic features. Interpretation indicated the biomechanical or clinical meaning inferred from the observations (e.g., malocclusion classification or treatment need). Biomechanics represented the recognition and evaluation of orthodontic appliances and their potential biomechanical function. Confidence denoted the model’s self-assigned certainty score on a scale from 0.0 to 1.0.

**Figure 2:**
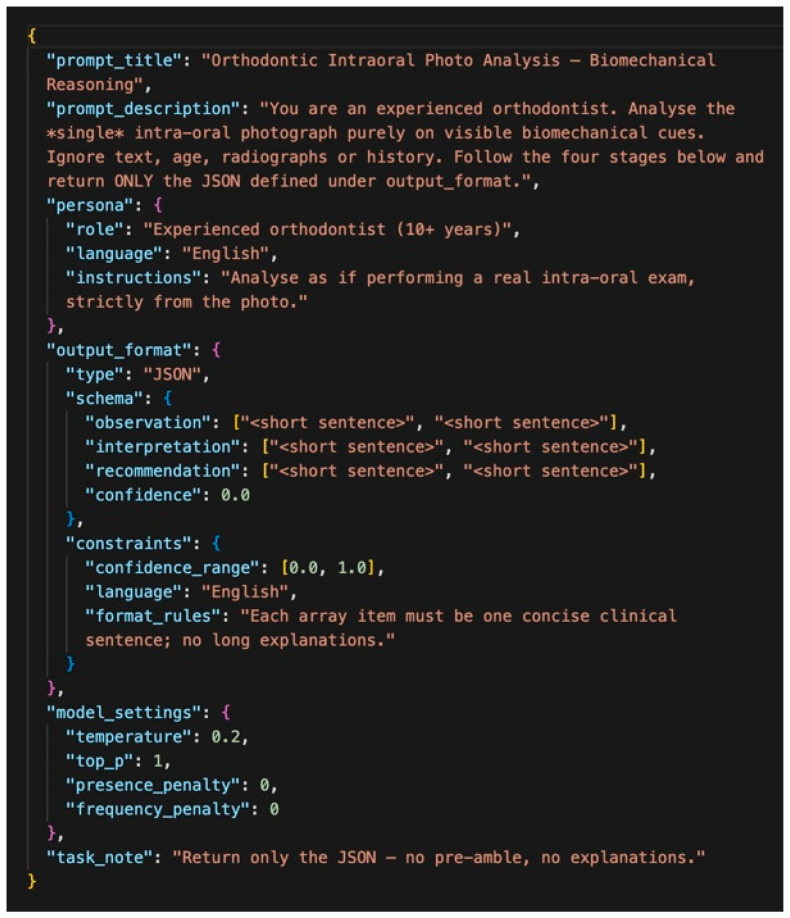
JSON-based structured prompt used to evaluate the biomechanical reasoning performance of LLMs. The prompt defines four output fields: observation, interpretation, biomechanics, and confidence. Only image-based reasoning is permitted; no external data are used.

This structured design enabled objective comparison of model outputs and facilitated reproducible expert evaluation. By constraining responses to predefined fields, the framework minimized ambiguity, reduced variability across models, and provided a clinically relevant basis for benchmarking biomechanical reasoning performance. The standardized JSON prompt used to elicit these outputs is illustrated in Figure 2. Details of the API call structure and image encoding protocol are provided in Section API Call Structure and Image Encoding, and the complete prompt text is available in Supplementary Material S1.

#### API Call Structure and Image Encoding

**Figure 3:**
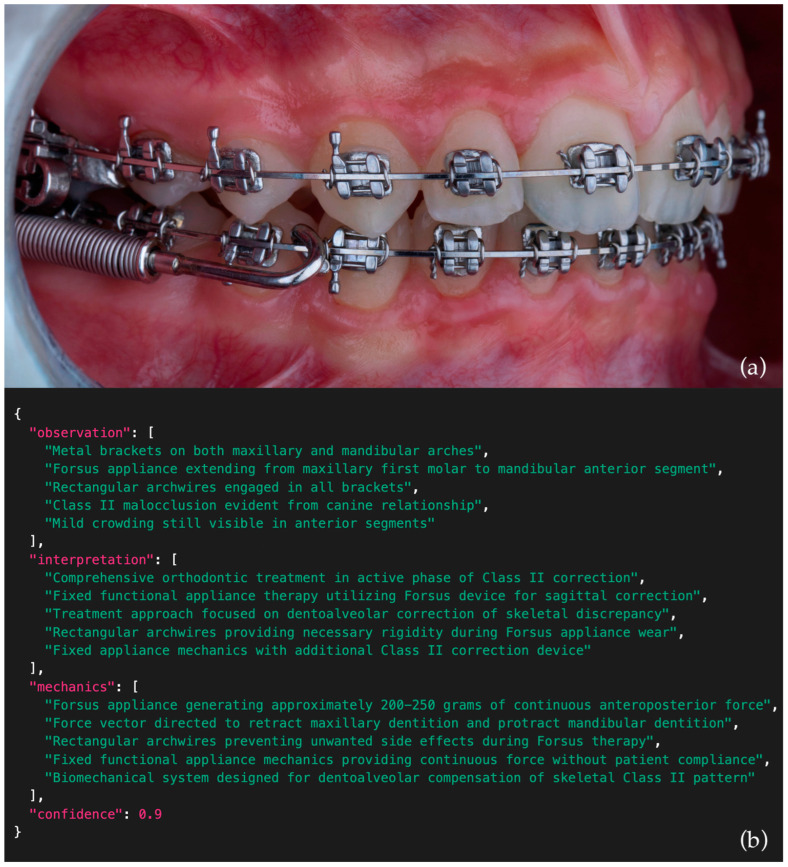
Example intraoral side-view photograph (**a**) and corresponding JSON output (**b**) generated by Claude 3.7 Sonnet (Anthropic). Some parts of the JSON output may not be fully visible due to scroll limitations in the original interface.

Each evaluation followed a standardized API call structure to ensure consistency across all models. After preprocessing, images were encoded in base64 format and embedded within JSON-structured requests. The API call comprised three components:

(1) a system message defining the evaluation task and output format,
(2) a user message containing the base64-encoded image and the structured prompt shown in Figure 2, and
(3) response formatting instructions specifying JSON output with four required fields.

For OpenAI (GPT-4.0, GPT-o3), images were submitted via the image URL field using a data-URI. For Anthropic (Claude 3.7), images were submitted as base64-encoded sources per provider documentation. All requests used the HTTP POST method with appropriate authentication headers. No additional image manipulation occurred after the initial JPEG conversion and base64 encoding.

The complete text of the system and user prompts is provided in Supplementary Material S1 to facilitate reproducibility and replication of the evaluation workflow.

### 2.4. Expert Evaluation

A panel of three experienced orthodontists independently evaluated the outputs generated by each model for all 56 images. The evaluators were blinded to model identity to prevent potential bias. Using a predefined Orthodontic Visual Assessment Rubric, each expert assigned scores from 1 (poor performance) to 5 (excellent performance) across four domains: observation, interpretation, biomechanics, and confidence evaluation.

The confidence evaluation domain reflected whether the model’s self-reported confidence score was appropriate, meaningful, and consistent with the quality of its overall output. Scoring in the other domains was based on the accuracy, completeness, and clinical relevance of the AI-generated responses. A composite performance score was calculated by averaging the four expert ratings for each model output.

**Figure 4:**
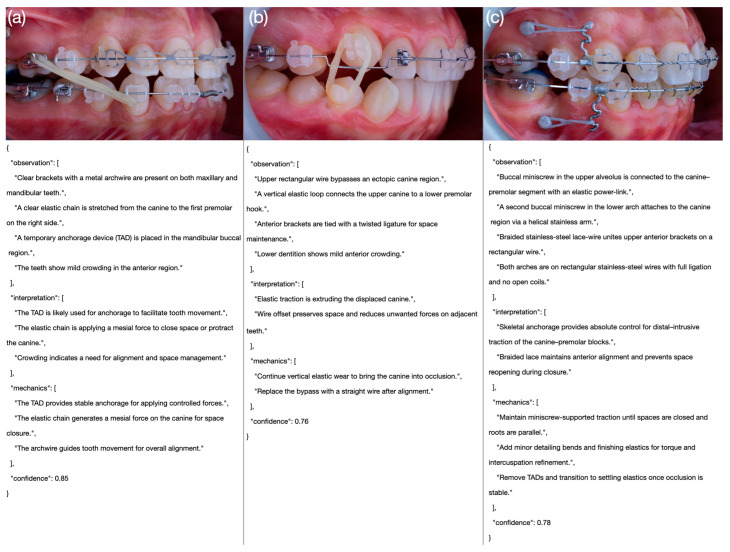
Representative model outputs across performance levels. (**a**) Grok 1.5 (1/5), (**b**) Gemini 2.5 (3/5), and (**c**) GPT-o3 (5/5). Each panel shows the intraoral image (**top**) and the corresponding JSON output (**bottom**).

Each score level (1–5) was operationally defined based on accuracy, completeness, and clinical reasoning quality, including representative examples for each domain and performance level. To ensure methodological transparency and reproducibility, the full scoring rubric—including detailed domain definitions, representative examples, and rating criteria—is provided in Table 3. An example intraoral side-view photograph together with the corresponding JSON output generated by one of the models is presented in Figure 3, while comparative examples of poor, moderate, and excellent model outputs are illustrated in Figure 4.

### 2.5. Error Analysis Protocol

To complement quantitative scoring, a qualitative error analysis was conducted to identify recurrent reasoning failures across biomechanical scenarios. All expert raters jointly reviewed representative model outputs for each complexity category (simple, moderate, complex) to characterize distinct error types. The analysis focused on identifying recurring patterns such as auxiliary misidentification, incorrect interpretation of force vectors, incomplete detection of posterior appliances, and overconfident predictions in ambiguous configurations.

### 2.6. Statistical Analysis

All statistical analyses were conducted using Jamovi (version 2.6.26.0; Sydney, Australia). Descriptive statistics were calculated to summarize model performance across evaluation domains. The Shapiro–Wilk test confirmed non-normality of the score distributions.

Inter-rater reliability among the three orthodontic evaluators was assessed using intraclass correlation coefficients (ICC [1,3], two-way mixed, absolute agreement), complemented by Cronbach’s alpha as a measure of internal consistency. Between-model comparisons were performed using Kruskal–Wallis tests, followed by Dwass–Steel–Critchlow–Fligner post hoc analyses for pairwise comparisons. Effect sizes were reported as eta-squared (ε^2^).

To account for the hierarchical structure of the data—multiple ratings nested within evaluators and images—linear mixed-effects models (LMMs) were also applied as a confirmatory analysis, including fixed effects for model type and evaluation domain, and random intercepts for evaluator and image. This approach ensured that non-independence among repeated ratings was properly controlled.

Additional analyses included multiple linear regression and exploratory factor analysis to examine score structures, as well as Spearman correlation to assess associations between model-reported confidence values and expert-rated confidence evaluations. Statistical significance was set at *p* < 0.05.

For each model output, a composite score was calculated by averaging the four expert ratings (observation, interpretation, biomechanics, and confidence evaluation). This composite metric reflects the overall strength of biomechanical reasoning within the broader clinical context, rather than the biomechanics domain alone, and was used for group-level comparisons and summary analyses. Calibration quality was assessed using the Expected Calibration Error (ECE) and Brier scores to evaluate the alignment between predicted confidence and expert-rated accuracy.

## 3. Results

### 3.1. Overall Model Performance

**Figure 5:**
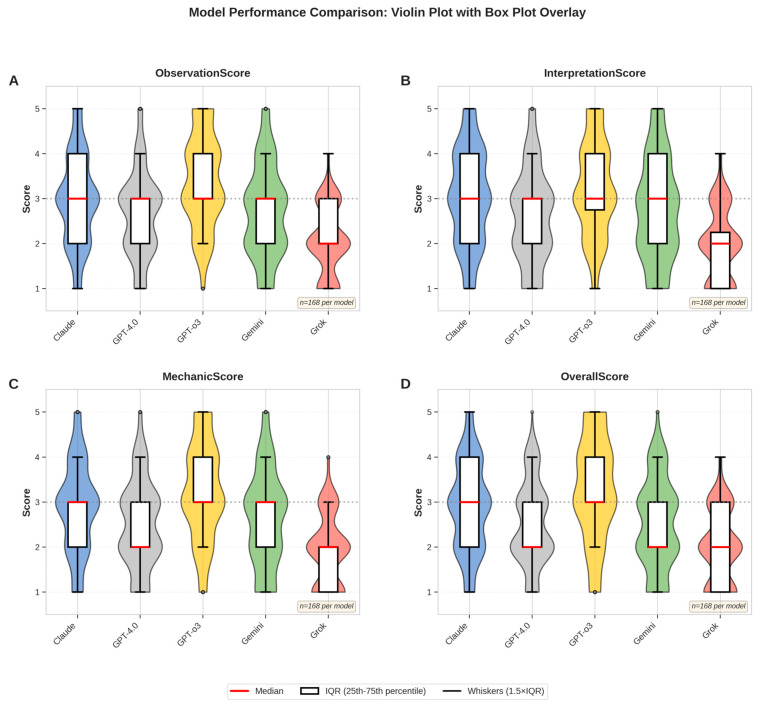
Comprehensive model performance comparison across all scoring dimensions. (**A**) ObservationScore, (**B**) InterpretationScore, (**C**) MechanicScore, and (**D**) OverallScore. Violin plots display the full distribution of expert ratings for each AI model, with overlaid box plots indicating the median (red line), interquartile range (white box), and whiskers extending to 1.5× the IQR. Individual outliers are shown as gray circles. The dashed horizontal line represents the midpoint (3.0) of the five-point Likert scale. Statistically significant differences were observed across all models (Kruskal–Wallis test, *p* < 0.001). n = 168 evaluations per model (56 images × 3 independent raters).

A total of 56 standardized intraoral side-view photographs were evaluated by three experienced orthodontists across five multimodal large language models (LLMs). Each evaluator rated the outputs across four domains—observation, interpretation, mechanic, and confidence evaluation—resulting in 840 complete assessments. After verification, no missing entries remained. Descriptive analyses revealed clear performance differences among the models. GPT-o3 consistently achieved the highest composite mean score (3.34/5.00; 66.8%), while Grok obtained the lowest (1.94/5.00; 38.8%). Claude, Gemini, and GPT-4.0 scored intermediately with means of 2.89 (57.8%), 2.63 (52.6%), and 2.44 (48.8%), respectively. A domain-level breakdown of scores is visualized in Figure 5, which illustrates score distributions using violin plots with overlaid box plots.

Descriptive statistics for each LLM are presented in Table 4. GPT-o3 achieved the highest mean scores across all expert-rated categories, followed by Claude and Gemini. Non-parametric Kruskal–Wallis tests confirmed significant differences among the models for all evaluation dimensions (*p* < 0.001). Post hoc Dwass–Steel–Critchlow–Fligner comparisons (Table 5) further indicated that GPT-o3 significantly outperformed all other models (*p* < 0.001), while differences between Claude and Gemini were not statistically significant.

### 3.2. Error Characterization and Performance Stratification

All 56 intraoral cases were grouped into three biomechanical complexity levels: simple, moderate, and complex (Table 6). Model performance declined progressively with increasing complexity. Common error patterns included (1) auxiliary misidentification, (2) force-vector misinterpretation, (3) incomplete detection of posterior appliances, and (4) overconfident but implausible predictions.

GPT-o3 and GPT-4.0 produced the most anatomically consistent results, whereas Claude and Grok showed frequent directional and detection errors. Performance degradation appeared driven mainly by spatial reasoning demands rather than visual noise.

Linear mixed-effects modeling confirmed significant effects of model type (χ^2^(4) = 142.6, *p* < 0.001) and evaluation domain (χ^2^(3) = 38.4, *p* < 0.001), with a model × domain interaction (χ^2^(12) = 28.7, *p* = 0.004). Variance analysis indicated that 78% of total variance originated from case-related differences, 2% from evaluator variance, and the remainder from residual sources, suggesting that most variability arose from image-level biomechanical complexity rather than rater inconsistency.

### 3.3. Inter-Rater Reliability

**Figure 6:**
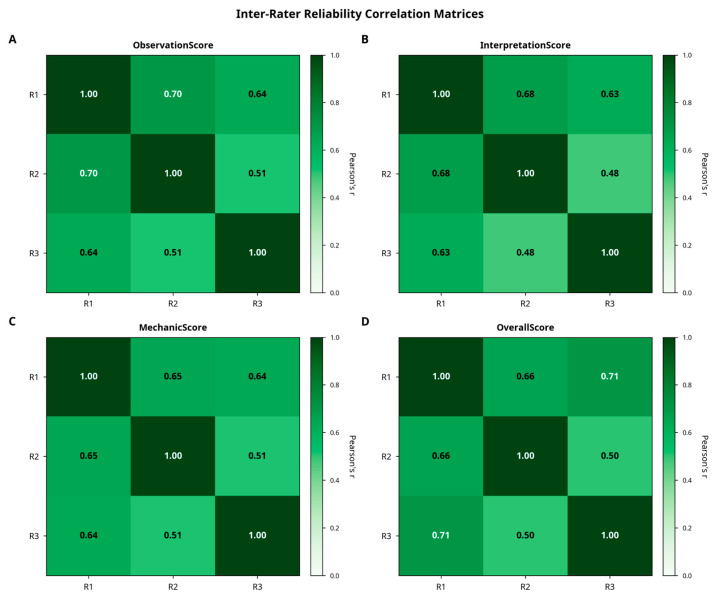
Inter-rater reliability correlation matrices. Heatmaps illustrate Spearman’s rank correlation coefficients between three independent raters for (**A**) ObservationScore, (**B**) InterpretationScore, (**C**) MechanicScore, and (**D**) OverallScore. All correlations were statistically significant (*p* < 0.001), indicating substantial agreement among raters across all scoring dimensions. Darker shades correspond to stronger correlations.

Inter-rater agreement was high across all scoring categories. Cronbach’s alpha ranged from 0.811 to 0.830, indicating strong internal consistency. Intraclass correlation coefficients (ICC [1,3], two-way mixed, absolute agreement) confirmed excellent reliability, with values ranging from 0.786 (Confidence Evaluation) to 0.802 (Observation). Variance decomposition indicated that approximately 78% of the total variance was attributable to case-related differences, while evaluator-related variance accounted for only 2%, highlighting the consistency of expert scoring. These relationships are illustrated in Figure 6.

### 3.4. Between-Model Comparisons

**Figure 7:**
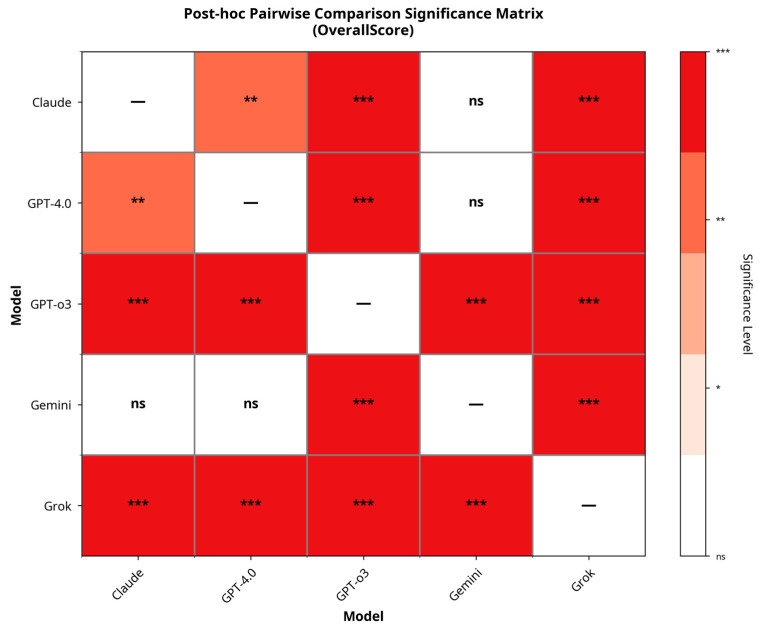
Pairwise comparison significance matrix for overall performance. *** *p* < 0.001, ** *p* < 0.01, *p < 0.05, ns = not significant.

A Kruskal–Wallis test revealed statistically significant differences in composite performance scores among the five models (χ^2^ = 156, df = 4, *p* < 0.001). Post hoc analyses with the Dwass–Steel–Critchlow–Fligner method showed that GPT-o3 significantly outperformed all other models (*p* < 0.001), while Grok scored significantly lower than all comparators. Claude performed significantly better than GPT-4.0 and Grok (*p* = 0.001 and *p* < 0.001, respectively) but did not differ from Gemini (*p* = 0.146). GPT-4.0 and Gemini also showed no significant difference (*p* = 0.662). Pairwise significance patterns are visualized in Figure 7.

### 3.5. Alignment Between Model Confidence and Expert Evaluation

To examine whether model-reported confidence scores aligned with expert assessments, a Spearman correlation analysis was performed. Across all outputs, a weak but statistically significant negative correlation was observed between self-assigned confidence and expert-rated confidence evaluation (ρ = −0.135, *p* = 0.023).

**Figure 8:**
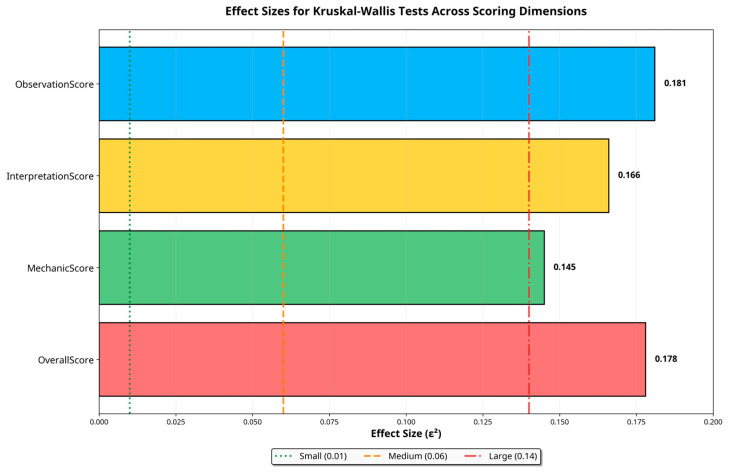
Effect sizes (ε^2^) derived from Kruskal–Wallis tests across scoring dimensions. All dimensions exhibit medium to large effect sizes, confirming substantial model performance differences.

When analyzed per model, Claude was the only system to demonstrate a significant positive correlation (ρ = 0.266, *p* = 0.047). GPT-o3, despite achieving the highest overall performance, exhibited a non-significant but positive trend (ρ = 0.159, *p* = 0.242). These findings indicate that high model confidence does not consistently reflect high expert-rated plausibility. Effect size comparisons for all scoring dimensions are shown in Figure 8.

### 3.6. Underlying Structure and Predictors of Expert Scoring

Exploratory factor analysis (minimum residual extraction, oblimin rotation) showed that all four scoring domains loaded strongly onto a single latent factor (factor loadings 0.873–0.990), suggesting that experts relied on a shared interpretive framework. Multiple linear regression demonstrated that the composite score was systematically composed of its subdomains, with 91.3% of its variance explained by observation, interpretation, and biomechanics (R^2^ = 0.913, F (3836) = 2930, *p* < 0.001). Among these, biomechanical reasoning contributed most strongly (β = 0.460), followed by observation (β = 0.292) and interpretation (β = 0.267).

### 3.7. Confidence Calibration Analysis

Model-reported confidence values were extracted directly from each API’s native probability or confidence field, where available. For models without explicit numeric confidence outputs, normalized proxy scores (0–1 scale) derived from textual self-confidence statements were independently verified by two evaluators for consistency. To evaluate whether model-reported confidence values accurately reflected expert-rated output quality, a calibration analysis was performed using the Expected Calibration Error (ECE) and Brier scores. Model outputs were grouped into five confidence bins (0.0–0.2, 0.2–0.4, 0.4–0.6, 0.6–0.8, and 0.8–1.0). Within each bin, the mean expert-rated accuracy was compared with the mean self-reported confidence.

Calibration metrics are summarized in Table 7. Lower ECE and Brier scores indicate better calibration. Among the evaluated models, Claude 3.7 demonstrated the best alignment between confidence and actual performance (ECE = 0.15; Brier = 0.21), followed by GPT-o3 (ECE = 0.18; Brier = 0.23). In contrast, GPT-4.0 and Grok 1.5 exhibited notable overconfidence, reporting higher mean confidence than warranted by their accuracy. Gemini 2.5 Pro showed moderate miscalibration with slightly inflated confidence values.

These results indicate that model-reported confidence scores are not fully reliable as standalone indicators of reasoning accuracy. For clinical use, confidence values should be interpreted cautiously and always validated through expert review rather than used as direct surrogates for diagnostic certainty.

### 3.8. Summary of Findings

Collectively, these findings demonstrate substantial variability in the ability of multimodal large language models to generate clinically coherent reasoning based solely on visual input. GPT-o3 not only achieved the highest absolute and relative performance but also exhibited the most consistent inter-expert agreement. These outcomes reinforce the value of structured visual assessment frameworks for benchmarking AI performance in orthodontic biomechanical reasoning and for supporting clinically meaningful decision-making.

## 4. Discussion

### 4.1. Principal Findings

The results of this study demonstrated substantial variation in biomechanical reasoning capabilities among the evaluated LLMs. GPT-o3 consistently achieved the highest scores across all domains, with superior recognition of orthodontic appliances and stronger biomechanical interpretation. In contrast, Grok performed poorly in both surface-level identification and deeper clinical logic, while Claude and Gemini achieved intermediate performance with outputs that were often correct in appliance detection but inconsistent in biomechanical reasoning. These differences were both statistically and clinically meaningful; GPT-o3’s mean score was approximately 75% higher than Grok’s, highlighting the impact of model choice on the reliability of AI-assisted orthodontic interpretation.

A notable finding was the misalignment between self-assigned confidence and expert-rated plausibility. Claude showed a significant positive correlation, whereas GPT-4.0 and Grok showed none. GPT-o3, despite its superior performance, reported only moderate confidence levels, indicating an “underconfident yet accurate” pattern that may be preferable in clinical contexts, where unjustified certainty could be misleading.

The performance differences observed among models likely stem from how each system processes and links visual and textual information. While all LLMs can recognize spatial patterns such as brackets or wires, they lack orthodontic domain knowledge needed to infer the mechanical purpose of these elements. As a result, some models may correctly identify appliances but misinterpret their biomechanical function. These findings suggest that model architecture and internal alignment between image and language representations play a key role in determining reasoning accuracy.

### 4.2. Comparison with Previous Studies

Several recent studies have explored LLMs for clinical image interpretation, particularly in radiology and dermatology. However, applications to intraoral imagery remain limited and often lack structured clinical validation. Moor et al. highlighted the broad capabilities of foundation models across diverse medical tasks, including imaging, but did not include structured expert validation or domain [21]. In contrast, our study used standardized prompts specific to orthodontic biomechanics and incorporated blinded expert assessments. Vassis et al. evaluated GPT-3.5 and GPT-4.0 for orthodontic patient education and found that while GPT-4.0 achieved higher content validity, it was still only partially satisfactory; nonetheless, 80% of patients preferred AI-generated content over traditional materials [30]. Horiuchi et al. reported that GPT-4.0 reached 43% diagnostic accuracy with text-based imaging descriptions, while GPT-4.0 scored only 8% when analyzing raw visual input [31]. In comparison, GPT-o3 achieved a mean expert score of 3.37 out of 5.00 (67.4%) in our study, highlighting recent progress in multimodal biomechanical reasoning.

Traditional AI models in dentistry, such as CNNs, have relied on labeled datasets for narrow tasks like Angle classification or anomaly detection [12,20]. While effective, they are often criticized as black-box systems with limited insight into how predictions are formed. In contrast, the LLMs evaluated in this study were not specifically trained for orthodontic tasks but demonstrated varied reasoning capabilities when guided with structured visual prompts. This observation is consistent with recent evidence summarized by Puleio et al. [23], who reviewed the clinical, research, and educational applications of ChatGPT across dental disciplines and emphasized the growing importance of domain-specific and explainable AI frameworks in dentistry. Because tools like SHAP [32] and LIME [33] require access to internal model components—such as gradients or attention layers—which are unavailable in most hosted multimodal systems [34], we adopted an external evaluation approach. This involved using standardized JSON prompts and expert scoring to assess interpretability. As described by Panda et al. [35] and Guo et al. [36], this strategy aligns with emerging principles in explainable AI (XAI) [37], which prioritize structured task design and human-centered evaluation over internal model inspection.

Traditional CNNs achieve high diagnostic accuracy but offer limited interpretability in biomechanical reasoning [12,20]. The present study prioritized clinical interpretability and structured reasoning over numerical accuracy, reflecting the complementary rather than competitive role of LLMs in orthodontic AI research. Unlike CNNs, which are trained on large, labeled image datasets for single-output classification, multimodal LLMs can integrate textual and visual information to generate stepwise biomechanical explanations. This makes them particularly suitable for analyzing orthodontic force systems, where understanding causal relationships and mechanical intent is more clinically relevant than pixel-level precision.

Another important challenge in using LLMs for clinical reasoning is hallucination when models generate fluent but inaccurate or misleading outputs. This risk increases with vague or underspecified prompts. Recent studies have shown that prompt specificity, internal consistency checks, and role-based task framing can help reduce such errors [38,39]. Informed by this, our study used structured prompts with clearly defined biomechanical expectations, which improved both output consistency and expert interpretability. Rather than depending solely on internal transparency, our approach treated explainability as a function of prompt structure and output control by emphasizing practical oversight as a key component of safe and interpretable clinical AI.

### 4.3. Clinical Implications

While these findings highlight the growing role of general-purpose LLMs in clinical image interpretation, prior CNN-based studies in dental AI provide valuable context. Bardideh et al. reported that AI models outperformed clinicians in Angle classification from intraoral photographs but were less accurate in estimating overjet and overbite [20]. Ryu et al. achieved >92% accuracy in predicting extraction decisions and crowding severity using CNNs trained on standardized buccal images [40]. Likewise, Ragodos et al. showed that a ResNet-18 model accurately detected anomalies such as hypoplasia and microdontia, supported by saliency maps [12]. Collectively, these studies confirm the high predictive power—but limited transparency—of black-box models. Although our study did not directly measure diagnostic accuracy, it extends this evidence by assessing the interpretive reasoning of LLMs through structured prompts and blinded expert scoring, bridging model output with clinical judgment.

These findings have important implications for orthodontic practice as remote monitoring and AI-assisted treatment planning become more common. Models capable of generating interpretable and clinically coherent reasoning are essential for safe integration. The structured, expert-based framework used here provides a reproducible method for evaluating such capabilities across LLMs. Among the tested models, only GPT-o3 consistently aligned with expert reasoning, whereas others—particularly Grok—produced less reliable outputs. Correlation analyses showed that model confidence did not consistently match expert-rated plausibility: Claude displayed a significant positive correlation, GPT-o3 showed a moderate but non-significant trend, and GPT-4.0 and Grok showed none. This underconfident yet accurate behavior of GPT-o3 may be preferable in clinical contexts, where unjustified certainty can be misleading. Overall, improved calibration of internal confidence scoring is needed to enhance transparency and trust in AI-assisted interpretation.

In practical orthodontic applications, this suggests that models capable of producing interpretable and clinically coherent reasoning will be critical for safe integration into workflows such as clinical decision-making, education, and adjunctive support tools, as well as remote monitoring and treatment planning. Among the evaluated systems, GPT-o3 demonstrated reasoning patterns most closely aligned with expert expectations, while others—particularly Grok—struggled to provide reliable outputs. Importantly, calibration of internal confidence scores remains necessary to avoid misleading certainty. Reliable clinical decision support will depend not only on accuracy but also on proper alignment between model confidence and expert plausibility.

Beyond clinical applications, the structured reasoning produced by multimodal LLMs can also support education. Their stepwise biomechanical explanations enable use in orthodontic teaching and calibration exercises, allowing trainees to compare their interpretations with AI-generated reasoning under expert guidance. Such integration may help standardize evaluation, foster critical analysis, and strengthen understanding of biomechanical principles in training environments [23].

This study provides a foundation for both clinical decision-making and educational innovation in orthodontics. By benchmarking LLMs through expert-rated biomechanical reasoning, it supports the development of interpretable and scalable AI systems that can assist clinicians, particularly in remote monitoring. The API-based, image-only framework allows integration into clinical workflows without additional infrastructure or fine-tuning, enhancing accessibility and transparency. As AI becomes more embedded in healthcare, structured and expert-validated evaluation frameworks like this one will be essential for responsible, patient-centered adoption.

### 4.4. Clinical Implementation: Thresholds, Workflows, and Regulatory Considerations

Translating these findings into clinical orthodontic practice requires defined accuracy thresholds, safe integration workflows, and regulatory awareness. A minimum composite score of 4.0/5.0 (≥80%) may represent a reasonable benchmark for clinical reliability in decision-support applications. At present, only GPT-o3 approaches this level in simple biomechanical cases, whereas all models perform below the desired threshold for complex scenarios. A supervised, clinician-guided workflow, in which AI systems perform preliminary image screening and orthodontists validate all outputs before clinical decisions, provides a practical and safe framework for real-world implementation.

Economic and regulatory considerations will shape future adoption. Although approximate API usage ranges are known, these values were not systematically analyzed or validated within the current study; therefore, cost estimations should be interpreted qualitatively rather than quantitatively. Current API pricing indicates modest usage costs, but financial feasibility depends on reimbursement policies and demonstrated improvements in patient outcomes. Systems offering diagnostic or treatment recommendations may qualify as Software as a Medical Device (SaMD) under FDA or EU MDR regulations, requiring validated accuracy, transparent documentation, and post-market monitoring to ensure patient safety in clinical deployment.

### 4.5. Limitations and Future Directions

This study has several limitations that should be considered when interpreting the findings. None of the evaluated models were fine-tuned for orthodontic applications, which may have contributed to variability in reasoning performance. The dataset was limited to standardized side-view intraoral photographs captured under controlled clinical conditions, and performance may differ under real-world imaging variability or with other view types. Moreover, evaluation was based on expert-graded reasoning quality rather than objective clinical outcomes, and API costs were not analyzed, despite being an important factor for scalability and accessibility. Future studies should incorporate cost–benefit analyses and explore model calibration across larger, more diverse datasets.

Model performance was inversely correlated with biomechanical complexity. While simple mechanics involving single appliances and straightforward force systems were well-interpreted by GPT-o3 and Claude, complex configurations with multiple auxiliaries and three-dimensional forces produced frequent reasoning errors across all models. Typical failure modes included auxiliary misidentification, misinterpretation of elastic force vectors, and incomplete detection of posterior appliances. These observations underscore the limited biomechanical abstraction capability of current multimodal LLMs when applied to realistic orthodontic scenarios.

Future research should focus on domain-specific fine-tuning, integration of multimodal data (photographic, radiographic, and textual), and benchmarking against clinically validated outcomes. Expanding datasets to include diverse imaging conditions and real-world variability will be critical for ensuring generalizability. Furthermore, incorporating adaptive or real-time reasoning systems—such as wearable or augmented-reality-assisted platforms—could enhance the clinical translation of AI-assisted orthodontic workflows.

## 5. Conclusions

Among the evaluated multimodal LLMs, GPT-o3 consistently produced the most reliable and clinically coherent reasoning. Although current multimodal LLMs exhibit only moderate quantitative accuracy, their structured reasoning and interpretability position them as promising adjuncts for orthodontic biomechanics education, remote monitoring, and decision support. These models demonstrate emerging potential to enhance clinical consistency and assist less experienced practitioners in understanding biomechanical force systems. Continued development through domain-specific fine-tuning, calibration of internal confidence scoring, and expert validation will be essential to achieve clinically deployable precision comparable to established CNN frameworks and ensure their safe and effective integration into orthodontic workflows.

## Figures and Tables

**Table 1:** Detailed Dataset Composition.

Characteristic	Category	n	%
Dentition Stage	Permanent	32	57.1
	Mixed	18	32.1
	Deciduous	6	10.7
Malocclusion Class	Class I	24	42.9
	Class II	21	37.5
	Class III	11	19.6
Appliance Type	Stainless steel brackets	38	67.9
	Ceramic brackets	8	14.3
	Self-ligating brackets	6	10.7
	Removable appliances	4	7.1
Mechanics Complexity	Simple	18	32.1
	Moderate	24	42.9
	Complex	14	25.0
View Type	Left side-view	27	48.2
	Right side-view	29	51.8
Image Quality	Standardized clinical photography	56	100.0

**Table 2:** Overview of model configurations and API specifications for all evaluated multimodal LLMs.

Parameter	Specification
Model Versions	GPT-o3: o3 GPT-4.0: gpt-4-turbo-2024-04-09 Claude 3.7 Sonnet: claude-3-sonnet-20240229 Gemini 2.5 Pro: gemini-2.5-pro-preview-0409 Grok: grok-1.5
API Endpoints	OpenAI: https://api.openai.com/v1/chat/completions (accessed on 18 April 2025; see https://openai.com) Anthropic: https://api.anthropic.com/v1/messages (accessed on 10 March 2025; see https://www.anthropic.com) Google: https://generativelanguage.googleapis.com/v1beta/models (accessed on 25 March 2025; see https://deepmind.google) xAI: Provider-specific endpoint (accessed on 28 April 2025; see https://x.ai)
Evaluation Period	March–April 2025
API Parameters	temperature: 0.0 max_tokens: 2000 top_p: 1.0 All other parameters = default values
Session Management	Each image evaluated in a fresh session to prevent contextual carry-over
Subscription Type	Standard commercial API access (no research-grade or preferential tiers)

**Table 3:** Orthodontic Visual Assessment Rubric used for expert evaluation of model outputs.

Domain	Definition/Purpose	Score 1 (Poor Performance)	Score 3 (Moderate Performance)	Score 5 (Excellent Performance)
Observation	Assesses the model’s ability to identify anatomical and orthodontic features visible in the photograph.	Fails to identify key structures or confuses anatomical regions.	Identifies most key structures with minor omissions.	Accurately and comprehensively identifies all relevant anatomical and orthodontic features.
Interpretation	Evaluates the clinical meaning inferred from the observations (e.g., malocclusion classification or treatment need).	Interpretation wholly incorrect or nonsensical.	Interpretation partially correct but includes notable inaccuracies.	Clinically sound and accurate interpretation of all observed features.
Biomechanics	Assesses recognition and evaluation of orthodontic appliances and their potential biomechanical function.	Fails to recognize or incorrectly describes the biomechanical system.	Recognizes main components but provides a partially flawed analysis of the force system.	Accurately identifies all components and provides a correct, detailed analysis of the biomechanical force system.
Confidence Evaluation	Reflects whether the model’s self-reported confidence score was appropriate, meaningful, and consistent with the quality of its output.	Confidence completely misaligned with output quality (e.g., high confidence in poor response).	Confidence moderately aligned with output quality.	Confidence well-calibrated and accurately reflects output quality and limitations.

**Table 4:** Descriptive Statistics for All Scoring Dimensions Across Multimodal Large Language Models.

Score Type	Model	N	Mean	Median	SD	Min	Max	IQR	95% CI Lower	95% CI Upper
Observation	Claude	168	3.02	3.00	1.05	1	5	2.00	2.86	3.18
	GPT-4.0	168	2.56	2.50	0.995	1	5	1.00	2.41	2.71
	GPT-o3	168	3.43	4.00	1.05	1	5	1.00	3.27	3.59
	Gemini	168	2.74	3.00	1.09	1	5	2.00	2.58	2.91
	Grok	168	2.04	2.00	0.803	1	5	1.00	1.91	2.16
Interpretation	Claude	168	2.86	3.00	1.12	1	5	2.00	2.69	3.03
	GPT-4.0	168	2.37	2.00	1.04	1	5	1.00	2.21	2.53
	GPT-o3	168	3.29	3.00	1.15	1	5	2.00	3.12	3.47
	Gemini	168	2.61	2.00	1.18	1	5	1.00	2.43	2.79
	Grok	168	1.87	2.00	0.816	1	5	1.00	1.74	1.99
Mechanic	Claude	168	2.81	3.00	1.11	1	5	2.00	2.64	2.98
	GPT-4.0	168	2.42	2.00	1.04	1	5	1.00	2.26	2.58
	GPT-o3	168	3.28	3.00	1.21	1	5	2.00	3.09	3.46
	Gemini	168	2.58	2.00	1.14	1	5	1.00	2.41	2.76
	Grok	168	1.93	2.00	0.793	1	5	1.00	1.81	2.05
Overall	Claude	168	2.86	3.00	1.07	1	5	2.00	2.70	3.03
	GPT-4.0	168	2.43	2.00	0.926	1	5	1.00	2.29	2.58
	GPT-o3	168	3.37	3.00	1.15	1	5	1.00	3.19	3.54
	Gemini	168	2.60	2.00	1.08	1	5	1.00	2.43	2.76
	Grok	168	1.93	2.00	0.770	1	5	1.00	1.81	2.05

SD = standard deviation; IQR = interquartile range; CI = confidence interval. Each model was evaluated over N = 168 reasoning samples per score type (56 images × 3 raters).

**Table 5:** Kruskal–Wallis Test Results for Model Performance Comparison.

Score Dimension	χ^2^	df	*p*-Value	ε^2^ (Effect Size)
Observation Score	152	4	<0.001	0.181
Interpretation Score	140	4	<0.001	0.166
Mechanic Score	122	4	<0.001	0.145
Overall Score	149	4	<0.001	0.178

Note: All comparisons showed statistically significant differences (*p* < 0.001) across the five AI models. Effect sizes (ε^2^) indicate medium to large practical significance.

**Table 6:** Performance Stratification by Biomechanical Complexity.

Complexity Level	Description	n	GPT-o3	Claude	Gemini	GPT-4.0	Grok
Simple	Single appliance, straightforward forces	18	3.8 ± 0.4	3.4 ± 0.5	3.1 ± 0.6	3.0 ± 0.5	2.9 ± 0.7
Moderate	Multiple components, conventional forces	24	3.4 ± 0.5	3.2 ± 0.6	2.9 ± 0.7	2.8 ± 0.6	2.5 ± 0.8
Complex	Multiple auxiliaries, 3D forces	14	2.9 ± 0.6	2.5 ± 0.7	2.3 ± 0.8	2.1 ± 0.7	1.8 ± 0.9

Values represent mean composite scores ± standard deviation (Scale: 1–5).

**Table 7:** Confidence Calibration Metrics.

Model	ECE ↓	Brier Score ↓	Mean Confidence	Mean Accuracy	Calibration Pattern
GPT-o3	0.18	0.23	0.68	0.76	Underconfident
Claude 3.7	0.15	0.21	0.72	0.74	Well-calibrated
Gemini 2.5	0.22	0.28	0.75	0.68	Slightly overconfident
GPT-4.0	0.24	0.31	0.78	0.65	Overconfident
Grok	0.31	0.39	0.82	0.58	Highly overconfident

ECE = Expected Calibration Error; Brier Score = mean squared error of probabilistic predictions. ↓ indicates that lower values represent better calibration.

## Data Availability

The data presented in this study are available on reasonable request from the corresponding author. The data are not publicly available due to privacy and ethical restrictions related to patient confidentiality.

## Supplementary Materials

The following supporting information can be downloaded at: https://www.mdpi.com/article/10.3390/bioengineering12111165/s1, S1. Complete System and User Prompt.

## Abbreviations

The following abbreviations are used in this manuscript:

ABO	American Board of Orthodontics
AI	Artificial Intelligence
API	Application Programming Interface
CNN	Convolutional Neural Network
ECE	Expected Calibration Error
ICC	Intraclass Correlation Coefficient
ML	Machine Learning
LMM	Linear Mixed-Effects Model
LLM	Large Language Model
MDR	Medical Device Regulation
XAI	Explainable Artificial Intelligence
JSON	JavaScript Object Notation
LIME	Local Interpretable Model-Agnostic Explanations
SHAP	Shapley Additive Explanations
SaMD	Software as a Medical Device
